# Effect of Radiance-Dimmer Devices Simulating Natural Sunlight Rhythm on the Plasma Melatonin Levels and Anxiety and Depression Scores of the Submarine Personnel

**Published:** 2019-04

**Authors:** Khodabakhsh Ahmadi, Majid Hazrati, Mohammadjavad Ahmadizadeh, Sima Noohi

**Affiliations:** Behavioral Sciences Research Center, Baqiyatallah University of Medical Sciences, Tehran, Iran.

**Keywords:** *Circadian Rhythms*, *Light*, *Melatonin*, *Submarine*

## Abstract

**Objective:** Not perceiving circadian shifts of sunlight due to living in enclosed environments may have deleterious effects on mental health and plasma parameters. This study aimed to determine the effect of dim regulation on the submarine personnel of Iranian Navy forces by radiating devices according to natural circadian sunlight shifts. Also, this study aimed to investigate the impact of mimicking sunlight circadian by artificial radiance luminating devices on the serological and psychological measures of submarine personnel.

**Method** **:** Participants were randomly assigned to experimental and control groups in this non-randomized controlled trial. There were 26 participants in each group, and they were all male aged 21-29 years. Both groups were living in the submarine underground hall, with 120 meters 2 area with constant radiance with the same intensity. The experimental group had been given extra lighting devices with changing radiance intensity according to the natural sunlight circadian cycles. Plasma melatonin levels and depression and anxiety scores were determined before and after the experiment for both groups. Minnesota Multiphasic Personality Inventory (MMPI) and Cattell’s Anxiety Scale Questionnaire (IPAT) were used to measure depression and anxiety, respectively.

**Results: **Findings indicate that the plasma melatonin levels (-16.2±13.6 vs 8.0±9.3 mg/dL, respectively; p<0.001), depression scores (-6±6 vs 3.9±5.4, respectively; p<0.001), and anxiety scores (-1±1.2 vs 0.73±1.04, respectively; p<0.001) significantly reduced in the experimental group compared to the control group.

**Conclusion: **Using radiance dimmers, with a radiance intensity regularity according to the sunlight, is effective in improving psychiatric and plasma parameters and can be used in closed occupational environments such as underground environments and submarine halls.

Alternation between feelings of sleepiness, awareness, and behavioral patterns depends on signals of outer world, such as the day/night cycle or the rate and the intensity of environmental light (1). In experimental models, it has been demonstrated that exposure to constant light can lead to depressive and anxiety disorders (2). Several studies have demonstrated that adult germ-free mice showed reduced anxiety-like behavior in the elevated plus maze,the light/dark test (L/D), and the open field (OF), with the results showing increased exploration of typically aversive zones (open arms in EPM, light chamber in L/D box, and center of the (OF) (3). Also, the role of light–dark transition task on general motor behavior and coordination was demonstrated. In that study, mice showed antianxiety and antidepressant behavior after this task (4, 5).

In mammals, the circadian clock is believed to be resided in suprachiasmatic nucleus (SCN), which is localized in the anterior hypothalamus, where the circadian rhythm originates.

 The eye has also been proven to have its own circadian oscillator which works independently from SCN. However, there is no direct evidence about any mutual influence between the two rhythms. Nonetheless, it has been reported that some biochemical rhythms in the SCN were completely abolished by bilateral ocular enucleation in experimental models, indicating that the vision is necessary for a circadian rhythm in SCN (6). 

In mammals, the pineal gland is the source of circulating melatonin (7), and the plasma concentrations increase during the biologic night than the day. In short-term, in day and night shift workers, night-time plasma melatonin levels was reduced by the diurnal light exposure (8). Circadian disruption (day-night or light-dark) has been shown to contribute to physical and psychological dimensionsin human beings, however, direct evidence on the same relation in the submarine sailors is not available (9). Although the literature suggests high levels of anxiety and depression in the submarine personnel (10), to our knowledge, no published study has investigated the effects of simulating natural diurnal light cycles on the serological and psychological parameters in this population (11). Stress and mental disorders, especially posttraumatic stress disorder is higher in military personnel. In this study, plasma melatonin levels were examined in a series of submarine personnel who worked in enclosed environments with artificial lights to study the potential effects of daytime perception using artificial illuminating devices that simulated natural diurnal light intensity cycle on the plasma melatonin and depression and anxiety levels of the participants. 

## Materials and Methods

***Study Design & Participants***

Due to the occupation of the study participants, it was not possible to use randomization approach, so the study was designed using non-randomized controlled trial method. With regards to the selection criteria and the recruitment of naval personnel, this sampling approach is likely to have no bias. Participants were recruited among Iranian submarine personnel and from a submarine squadron. All Iranian marine forces working in the selected submarines were selected to participate in the study, as the marine forces are carefully selected. All participants were in good health based on their routine medical screenings; they were all male, and aged 21-29 years. Finally, 52 males working in a submarine squadron were included in the study and were divided into two groups based on their submarine assignment (26 in the experiment group, 26 in the control group). The study was conducted from 01/21/2016 to 01/20/2017. 

***Inclusion & Exclusion Criteria***

Inclusion criteria are as follow: (1) no major health problem such as: having significant acute or chronic systemic disorders, including type I diabetes mellitus, uncontrolled thyroid disorders, and kidney/prostate/bladder problems which cause excessive urination, congestive heart failure, angina pectoris, or other major cardiovascular disorders, hepatitis, asthma or severe respiratory allergies, stroke, Parkinson’s disease, Alzheimer’s disease, schizophrenia, epilepsy, oncologic disorders if less than 1 year had passed since the end of treatment, or chronic infectionsbased on physical examination and laboratory tests. ; (2) Exclusion criteria were as follow: (1) taking medications that could affect serum melatonin levels; (2) having depression or any other major psychiatric disorders; ; (3) Using hypnotics, melatonin, stimulants, or other medications that may affect melatonin levels (eg, β blockers, drugs that affect prostaglandin synthesis, or drugs that activate hepatic melatonin metabolism); (5) unwillingness or inability to discontinue the occasional use of these medications for at least 4 weeks prior to the study; and (6) any level of alcohol use or other substance abuse. 

***Sampling***

Randomization of the participants was not possible due to the strict military protocols. Participants in either group were comparable in their demographic features, including gender, age, and education level (p>0.9). The study population included two groups: 26 submarine personnel in the experimental and 26 in the control group. In this study, clinical interview with the study participants before the study revealed symptoms of depression and excessive strain during their working times.

***Ethics***

All the potential participants were kindly asked to participate in a session describing the study protocol, goals, and its potential positive or negative effects. All participants provided informed consent. Also, the necessary organizational coordination was done for the implementation of this study. This study was approved by the ethics committee of Baqiyatallah University of Medical Sciences (Code: IR.BMSU.REC.1395.232). 

***Laboratory Measures***

Serum samples were separated by centrifugation and stored at -20ºC. Melatonin concentrations were measured by radioimmunoassay (RIA) in duplicate 1 mL serum sample using CIDtech Ultraspecific Melatonin Antiserum (CID-tech Research Inc., Hamilton, Ontario). The assay procedure has been described in detail elsewhere (13). In brief, one mL serum sample was extracted into 5 mL of chloroform, the organic extracts were evaporated under a stream of nitrogen, and the remaining residues were dissolved in phosphate buffer. Samples of the buffered extracts of the serum were then analyzed by RIA. 

***Instruments***

1. Minnesota Multiphasic Personality Inventory (MMPI)

MMPI is a self-rating inventory with 566 descriptive statements, and respondents should answer to each of them by “Yes” or “No” (14). The MMPI is a comprehensive inventory measuring a vast number of clinical scales including hypochondriasis (32 items), hysteria (60 items), psychopathic deviate (50 items), masculinity/femininity (56 items), paranoia (40 items), psychasthenia (48 items), schizophrenia (78 items), hypomania (46 items), social introversion (69 items), and depression (57 items). In this study, depression subscale of MMPI was used. This subscale has 57 items for assessing depression levels. The scale was used at the beginning and after the intervention. Then, the crude scores were converted to scale T on a scale. Here, scores of 65 and more were indicative of clinical depression. 

2. Cattell and Schier’s Institute for Personality and Ability Testing's Anxiety Scale Questionnaire (IPAT)

The validated version of Cattell’s IPAT anxiety scale was employed to examine anxiety levels in submarine personnel before and after the study course. The raw scores for the five Cattell’s IPAT anxiety scales are transformed into norm scores and presented as follow: Cattell’s IPAT score of >seven indicated anxiousness, score of <four non-anxious, and 4-7 moderate anxiety. 

***Intervention Protocols***

The study was performed in a submarine squadron that belonged to Iranian Navy forces. The environment in which the personnel were serving was 120 m2 space with no natural light. They benefited from artificial light during six AM and 6 PM five days a week. To light the environment, 4 automatic optic devices were installed at the halls where the personnel from the experimental group were working. Two types of light (sunlight lamps and moonlight lamps), with radiant intensity were used (200 lux sunlight, and 200 moonlit). These optic devices were turned on at six AM automatically, which was the approximate time of sunrise. Also, during sunrise to adjust with the natural sunrise, 68 lux per hour was added to the intensity of radiation, so the intensity of the light regulators at 12 o'clock was close to its transparent proximity (400 deluxe for any regulator). Then, the intensity of its radiation was reduced by about 68 lux per hour, and as a result, the intensity of the light shut off at 6 o'clock in the afternoon simultaneously with the approximate time of sunset. Lighting was done only for the experimental group for three months, while the control group continued their experience with constant artificial light during the same period. After this time, the study variables were reanalyzed and compared to the baseline values.

***Statistical Analysis***

SPSS version 17.0 (SPSS Inc, Chicago, Il, USA) was used for data analysis. Chi square test was used to analyze categorical data and student’s t test to compare continuous data between the two groups. Correlation studies were performed using Pearson’s correlation test. A p value<0.05 was considered statistically significant.

## Results

The results showed that the plasma melatonin levels after the study decreased significantly in the experiment group versus the controls (64.9±17.7 vs 89.3±23.2 mg/dL, respectively; p<0.001). Also, changes in the plasma melatonin levels after the study (versus baseline levels) revealed the same relationship (-16.2±13.6 vs 8.0±9.3 mg/dL, respectively; p<0.001). (Table 1, line: 3, 4). 

**Figure 1:**
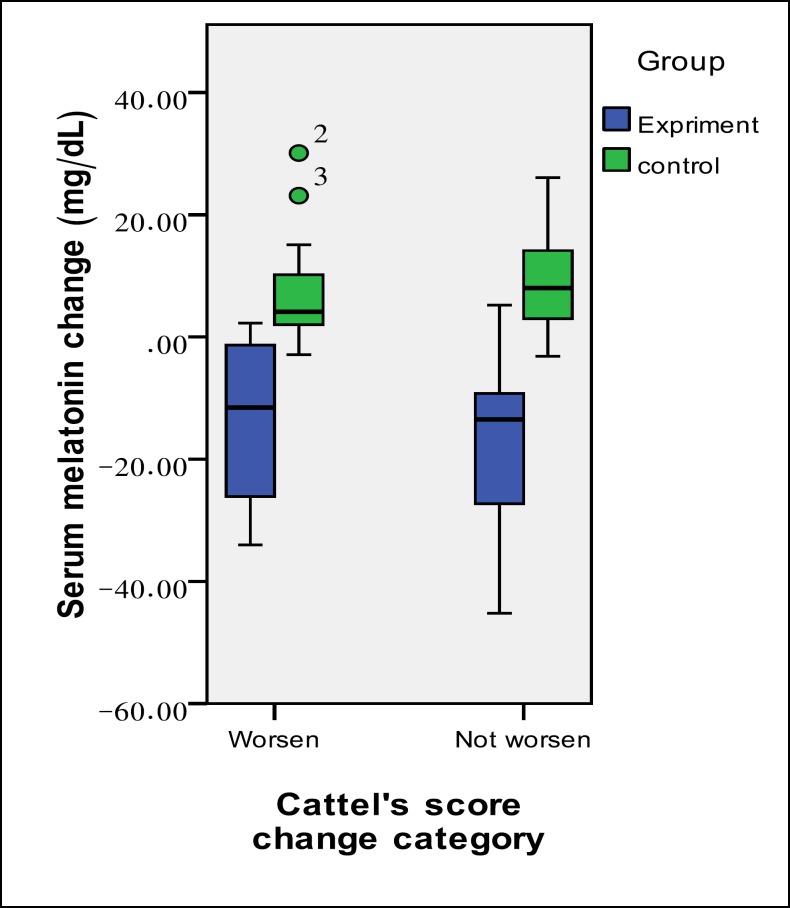
Change in Serum Melatonin Levels in the Study Groups According to the Change in their Anxiety Status

**Figure 2:**
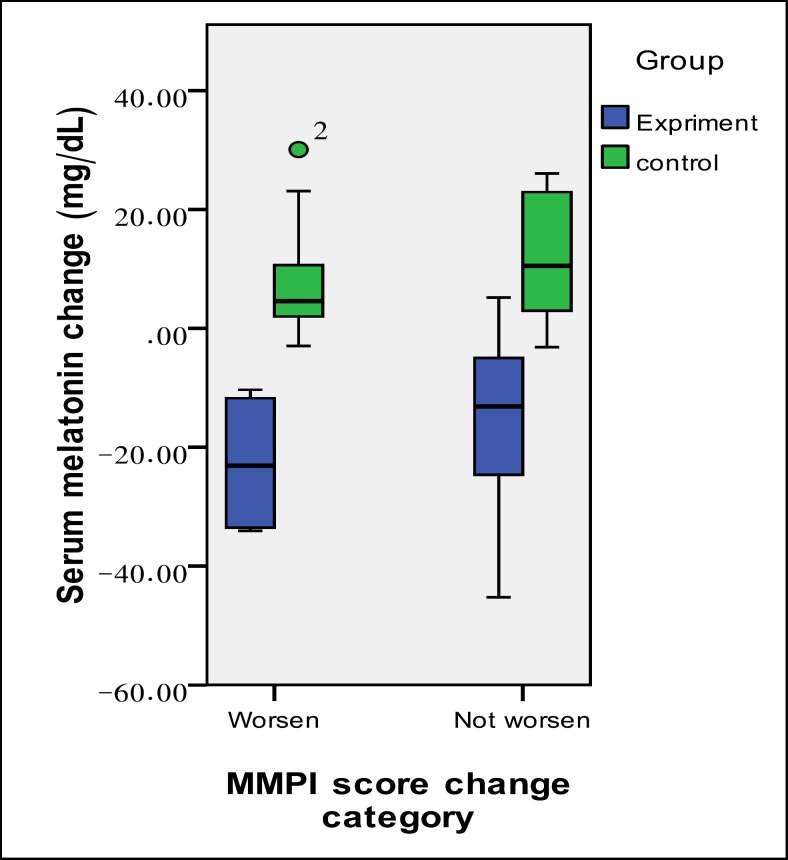
Change in Serum Melatonin Levels in the Study Groups According to the Change in their Depression Status

## Discussion

Findings of this study indicate that compared to usual constant lighting of the submarines, adding artificial radiance devices to the natural day and night cycles to the conventional constant lighting at the same environments significantly decreases plasma melatonin levels in the submarine personnel and significantly reduces the anxiety levels measured by Cattell’s IPAT and the self-rated depression levels measured by MMPI questionnaire in this population. The results of this study are consistent with the debates supporting the significant role of light intensity cycles correspondent to the day and night rhythms in regulating circadian cycles in either biological or psychological aspects.

**Table1:** T Test for Compare of Melatonin, Anxiety and Depression in Experiment and Control Groups

**Variable**	**Tests**	**Group**	**N**	**Mean**	**Std.Deviation**	**t**	**df**	**sig**
Melatonin	Pre test	Experiment	26	81.12	21.3	0.034	50	0.97
		Control	26	81.31	19.4			
	Post test	Experiment	26	64.89	17.7	4.26	50	0.0001
		Control	26	89.29	23.1			
Anxiety	Pre test	Experiment	26	3.62	1.6	0.318	50	0.75
		Control	26	3.46	1.8			
	Post test	Experiment	26	2.62	1.4	2.87	50	0.006
		Control	26	4.19	1.9			
Depression	Pre test	Experiment	26	63.23	10.6	1.70	50	0.094
		Control	26	58.46	9.4			
	Post test	Experiment	26	57.23	9.6	2.83	50	0.043
		Control	26	62.35	10.5			

Also, the lighting devices with periodic light intensity rhythms were not of such intensity that could affect the constant radiation intensity and relevance of the former can be attributed to providing the crew with the ability to estimate the approximate daytime by having a look at the device’s light intensity without looking at a clock or watch. The relevance of this issue mainly comes from the reports indicating altering effects of light intensity on the plasma levels of melatonin. 

Melatonin is synthesized from L-tryptophan (a dietary amino acid precursor) and its plasma levels depends on several factors, including the rate of its enzymatic formation by serotonin N-acetyltransferase (15), and to a lesser degree, to tryptophan hydroxylase (16,17), some nutritional factors (the availability of tryptophan (18), folate (19), and vitamin B6 (20,21)), demographic factors (age (22,23), blindness (24)), clinical illnesses (liver diseases (25), end-stage renal failure (26), cardiovascular diseases (27), diabetic autonomic neuropathy (28)), and psychiatric disorders (depression (29,30)), cancers (31,32), medications (33), and light intensity. The effect of melatonin on psychological disorders was such that several studies have investigated the impact of melatonin administration on improvement of these disorders and found positive effects (34). Tests of melatonin plasma levels were done in the morning.

Evidence strongly recommends that the level of radiance received by a given species is strongly predictive of plasma melatonin levels in a way that it would be high during the dark phase of any natural or imposed light-dark illumination cycle and vice versa, irrespective of its activity-time pattern, whether it is a night- or day-acting species (35-37). It is a most interesting example of an interaction of environmental factors with physiological processes especially regulating internal circadian rhythms. The relevance of the issue would hit a spike when we research the health and regulation of the circadian rhythms in people working in enclosed life environments like submarine personnel who do not receive natural light-dark circle provided by day and night circulation. It is also demonstrated that altered plasma melatonin levels can effectively affect several physiological factors like core body temperature (38), sleep health, and disturbances (39-41), psychiatric disturbances like depression (42,43). 

To date, several studies have reported altering effects of light and darkness on the melatonin plasma levels (44). However, to our knowledge, no study has investigated the potential effects of people’s perception of the day-time through looking at radiance devices with regulatory rhythmic light intensity changes based on natural day-night light circles on plasma melatonin levels or sleep and/or psychiatric status. 

They reported numbness, sleepiness, and boredom. Nonetheless, the personnel in the experiment group expressed significant improvement in their depression and anxiety scores, and also a significant decrease was detected in their serum melatonin levels. Personnel of the experimental group also reported that after the experiment, they could well guess the approximate time and hour only by looking at the intensity of the radiance of lighting devices without looking at their watch.

In the control group, however, the levels of melatonin and the psychological measures did not only decrease but also significantly increased during the study process. Some explanations can be given for this observation. It is already known that submarine service is associated with significant physical and psychological morbidity (45), therefore, it is understandable that following a research protocol may cause even more distress. On the other hand, the constant radiance provided in the submarine environment may be another explanation for the observed distress in the personnel due to disturbing their natural perception of the day-night cycle by the intensity of the sunlight. Even in the rainy areas in which clouds may obscure the natural sunlight for a long time, it has been demonstrated that people can develop excessive depression and anxiety levels compared to the cloudless days in which they can have the sunshine (46). Thus, adding this to living in an enclosed environment for a relatively long-time may have cumulative adverse effects on the personnel’s psychiatric scores. However, the personnel in the experimental group who were only given the possibility of having an approximate guessing of the daytime, had a significant decrease in their psychiatric disturbance scores and melatonin levels, despite receiving comparable intensity of constant radiance provided to the control group. One simple explanation can be the beneficial effects of recognizing the day time by visual perception rather than looking at the clock. However, another explanation may be that thinking that the personnel felt good because they thought their health is important to authorities. The latter explanation might be supported by a previous report in which only a training course for the submarine personnel was associated with a significant decrease in their anxiety score (47). 

## Limitation

Some of them were as follow: It was not possible to measure job status, mission duration, personality traits, or other factors such as Family. Also, demonstrating efficacy over a full year and determining the impact of different seasons were not possible. Other limitations were small number of participants and history of past sleep disorder.

## Conclusion

It was found that adding limited number of radiance devices simulating the sunlight intensity in the enclosed life environment of submarines have significant beneficial effects on the personnel’s psychiatric scores and serum melatonin levels. The reason(s) behind the observations of this study is still uncertain and needs clarification by conducting future studies.

## References

[1] Roenneberg T, Kantermann T, Juda M, Vetter C, Allebrandt KV (2013). Light and the human circadian clock. Handb Exp Pharmacol.

[2] Tapia-Osorio A, Salgado-Delgado R, Angeles-Castellanos M, Escobar C (2013). Disruption of circadian rhythms due to chronic constant light leads to depressive and anxiety-like behaviors in the rat. Behav Brain Res..

[3] Foster JA1, McVey Neufeld KA (2013). Gut–brain axis: how the microbiomeinfluences anxiety and depression. Trends Neurosci.

[4] Walf AA, Frye CA (2010). Estradiol reduces anxiety-and depression-like behavior of aged female mice. Physiol Behav.

[5] Arrant AE, Schramm-Sapyta NL, Kuhn CM (2013). Use of the light/dark test for anxiety in adult and adolescent male rats. Behav Brain Res..

[6] Beaulé C, Robinson B, Lamont EW, Amir S (2003). Melanopsin in the circadian timing system. J Mol Neurosci.

[7] He C, Wang J, Li Y, Zhu K, Xu Z, Song Y (2015). Melatonin-related genes expressed in the mouse uterus during early gestation promote embryo implantation. J Pineal Res.

[8] Papantoniou K, Pozo OJ, Espinosa A, Marcos J, Castaño-Vinyals G, Basagaña X (2014). Circadian variation of melatonin, light exposure, and diurnal preference in day and night shift workers of both sexes. Cancer Epidemiol Biomarkers Prev.

[9] Peng WG, Hong LB, Fang F (2002). EFFECT OF LONG TERM VOYAGE ON ANXIETY, DEPRESSION AND SLEEP QUALITY OF SAILORS IN NAVAL SHIP AND NUCLEAR-POWERED SUBMARINE. Journal of Preventive Medicine of Chinese People's Liberation Army..

[10] Chabal S, Welles R, Haran FJ, Markwald R (2018). Effects of sleep and fatigue on teams in a submarine environment. Undersea Hyperb Med.

[11] Anisi J, Majdian M, Mirzamani SM (2010). The factors associated with suicide ideation in Iranian soldiers. Iran J Psychiatry.

[12] Soltaninejad A, Fathi-Ashtiani A, Ahmadi K, Sadat Mirsharafoddini H, Nikmorad A, Pilevarzadeh M (2014). Personality Factors Underlying Suicidal Behavior Among Military Youth. Iran Red Crescent Med J.

[13] Brzezinski A, Seibel MM, Lynch HJ, Deng MH, Wurtman RJ (1987). Melatonin in Human Preovulatory Follicular Fluid. J Clin Endocrinol Metab.

[14] Iuvone PM, Tosini G, Pozdeyev N, Haque R, Klein DC, Chaurasia SS (2005). Circadian clocks, clock networks, arylalkylamine N-acetyltransferase, and melatonin in the retina. Prog Retin Eye Res.

[15] Klein DC (2007). Arylalkylamine N-acetyltransferase: “the Timezyme”. J Biol Chem..

[16] Cahill GM, Besharse JC (1990). Circadian regulation of melatonin in the retina of Xenopus laevis: limitation by serotonin availability. J Neurochem,.

[17] Thomas KB, Brown AD, Iuvone PM (1998). Elevation of melatonin in chicken retina by 5-hydroxytryptophan: differential light/dark responses. Neuroreport.

[18] Zimmermann RC, McDougle CJ, Schumacher M, Olcese J, Mason JW, Heninger GR (1993). Effects of acute tryptophan depletion on nocturnal melatonin secretion in humans. J Clin Endocrinol Metab.

[19] Fournier I, Ploye F, Cottet-Emard JM, Brun J, Claustrat B (2002). Folate deficiency alters melatonin secretion in rats. J Nutr..

[20] Luboshitzky R, Ophir U, Nave R, Epstein R, Shen-Orr Z, Herer P (2002). The effect of pyridoxine administration on melatonin secretion in normal men. Neuro Endocrinol Lett.

[21] Viswanathan M, Siow YL, Paulose CS, Dakshinamurti K (1988). Pineal indoleamine metabolism in pyridoxine-deficient rats. Brain Res.

[22] Magri F, Sarra S, Cinchetti W, Guazzoni V, Fioravanti M, Cravello L (2004). Qualitative and quantitative changes of melatonin levels in physiological and pathological aging and in centenarians. J Pineal Res.

[23] Wu YH, Swaab DF (2005). The human pineal gland and melatonin in ageing and Alzheimer’s disease. J Pineal Res.

[24] Skene DJ, Arendt J (2007). Circadian rhythm sleep disorders in the blind and their treatment with melatonin. Sleep Med.

[25] Steindl PE, Finn B, Bendok B, Rothke S, Zee PC, Blei AT (1995). Disruption of the diurnal rhythm of plasma melatonin in cirrhosis. Ann Intern Med.

[26] Lüdermann P, Zwernemann S, Lerchl A (2001). Clearance of melatonin and 6-sulfatoxymelatonin by hemodialysis in patients with end-stage renal disease. J Pineal Res.

[27] Yaprak M, Altun A, Vardar A, Aktoz M, Ciftci S, Ozbay G (2003). Decreased nocturnal synthesis of melatonin in patients with coronary artery disease. Int J Cardiol.

[28] Tutuncu NB, Batur MK, Yildirir A, Tutuncu T, Deger A, Koray Z (2005). Melatonin levels decrease in type 2 diabetic patients with cardiac autonomic neuropathy. J Pineal Res.

[29] Kennedy SH, Kutcher SP, Ralevski E, Brown GM (1996). Nocturnal melatonin and 24-hour 6-sulphatoxymelatonin levels in various phases of bipolar affective disorder. Psychiatry Res.

[30] Pacchierotti C, Iapichino S, Bossini L, Pieraccini F, Castrogiovanni P (2001). Melatonin in psychiatric disorders: a review on the melatonin involvement in psychiatry. Front Neuroendocrinol.

[31] Schernhammer ES, Hankinson SE (2005). Urinary melatonin levels and breast cancer risk. J Natl Cancer Inst.

[32] Schernhammer ES, Kroenke CH, Laden F, Hankinson SE (2006). Night work and risk of breast cancer. Epidemiology.

[33] Skene DJ, Bojkowski CJ, Arendt J (1994). Comparison of the effects of acute fluvoxamine and desipramine administration on melatonin and cortisol production in humans. Br J Clin Pharmacol.

[34] Costello RB, Lentino CV, Boyd CC, O'Connell ML, Crawford CC, Sprengel ML, Deuster PA (2014). The effectiveness of melatonin for promoting healthy sleep: a rapid evidence assessment of the literature. Nutr J.

[35] Skene DJ, Arendt J (2006). Human circadian rhythms: physiological and therapeutic relevance of light and melatonin. Ann Clin Biochem.

[36] Ho Mien I, Chua EC, Lau P, Tan LC, Lee IT, Yeo SC (2014). Effects of exposure to intermittent versus continuous red light on human circadian rhythms, melatonin suppression, and pupillary constriction. PLoS One.

[37] Reiter RJ (1991). Pineal melatonin: cell biology of its synthesis and of its physiological interactions. Endocr Rev.

[38] Rajaratnam SM, Arendt J (2001). Health in a 24-h society. Lancet.

[39] Kräuchi K, Cajochen C, Pache M, Flammer J, Wirz-Justice A (2006). Thermoregulatory effects of melatonin in relation to sleepiness. Chronobiol Int.

[40] Arendt J (2005). Melatonin: characteristics, concerns, and prospects. J Biol Rhythms.

[41] Scheer FA, Czeisler CA (2005). Melatonin, sleep, and circadian rhythms. Sleep Med Rev.

[42] Lewy AJ, Lefler BJ, Emens JS, Bauer VK (2006). The circadian basis of winter depression. Proc Natl Acad Sci U S A.

[43] Lewy AJ, Rough JN, Songer JB, Mishra N, Yuhas K, Emens JS (2007). The phase shift hypothesis for the circadian component of winter depression. Dialogues Clin Neurosci.

[44] Jasser SA, Hanifin JP, Rollag MD, Brainard GC (2006). Dim light adaptation attenuates acute melatonin suppression in humans. J Biol Rhythms.

[45] Kang J, Song YM (2017). The association between submarine service and multimorbidity: a cross-sectional study of Korean naval personnel. BMJ Open.

[46] Lambert G, Reid C, Kaye D, Jennings G, Esler M (2003). Increased suicide rate in the middle-aged and its association with hours of sunlight. Am J Psychiatry.

[47] Van Wijk C (1998). Submarine escape: the effect of training on anxiety. Mil Med.

